# Hodgkin Lymphoma Mimicking Inflammatory Breast Carcinoma: A Rare Case with Diagnostic Challenge and Novel Treatment

**DOI:** 10.1155/2019/9256807

**Published:** 2019-12-05

**Authors:** Eleni Thodou, Maria Befani, George Triantafyllidis, Theodosia Choreftaki, George Kanellis, Nikolaos Giannakoulas

**Affiliations:** ^1^Department of Pathology, Medical School, University of Thessaly, Viopolis, 41500 Larisa, Greece; ^2^Hematology, Private Practice, 61 Koumoundourou Str., 38221 Volos, Greece; ^3^Radiology, Private Practice, 29 Glavani Str., 38221 Volos, Greece; ^4^Department of Pathology, “G. Genimmatas”, Athens General Hospital, 154 Mesogeion Ave., 11527 Athens, Greece; ^5^Department of Hematopathology, “Evangelismos”, General Hospital, 45 Ypsilantou Str., 10676 Athens, Greece; ^6^Department of Hematology, Medical School, University of Thessaly, Viopolis, 41500 Larisa, Greece

## Abstract

Extranodal Hodgkin lymphoma involving the breast is infrequent. Most cases reported in the literature were diagnosed by histology after lumpectomy. We present a Hodgkin lymphoma mimicking inflammatory breast carcinoma in a 57-year-old woman. The diagnosis was performed by fine-needle aspiration (FNA) of the breast lesion and the axillary lymph nodes with rapid on-site evaluation followed by immunocytochemistry, and it was confirmed by histology. The patient after first-line chemotherapy developed relapse/refractory disease. Salvage chemotherapy regimens were applied with poor results and severe toxicity. Total remission was achieved with monotherapy of brentuximab vedotin, a novel anti-CD30-targeted antibody drug conjugate. This is a unique case of breast HL with misleading clinical presentation initially diagnosed by cytology. FNA as a minimally invasive diagnostic tool was crucial in avoiding unnecessary breast surgery and further delay of chemotherapy. It is also the first report highlighting the importance of this novel immunotherapy in the management of refractory Hodgkin lymphoma with breast involvement.

## 1. Introduction

Breast lymphomas account for less than 0.5% of all malignant breast tumors [1]. Particularly, in a large multicenter study, the prevalence of breast lymphoma has been defined at 1.6% among all cases with non-Hodgkin Lymphoma (NHL). Furthermore, primary breast lymphoma represents a small proportion of extranodal lymphomas ranging from 0.85% to 2.2% [2]. Primary breast lymphomas are usually NHL, in specific diffuse B large cell, Burkitt lymphoma, T anaplastic cell lymphoma, and mucosa-associated lymphoid tissue lymphoma [3].

Breast is an uncommon site of extranodal Hodgkin lymphoma (HL). Given that only sporadic cases of breast HL have been reported in the literature, the exact prevalence, either as a primary tumor or in the process of disseminated or recurrent disease, cannot be defined [4, 5].

The diagnosis of lymphomas is traditionally established by histology on a surgical biopsy material with an appropriate panel of immunostains. However, in the last decade, FNA has reached an important and definite role in the diagnosis of lymphomas and reactive lesions in both lymph nodes and extranodal sites with the use of ancillary techniques such as flow cytometry and immunocytochemistry. FNA assisted by flow cytometry can further contribute significantly in the subclassification of NHL [6]. Similarly, in lymphoid breast lesions, the combination of FNA cytology with these techniques can provide specific diagnoses, distinguishing reactive from neoplastic processes, with a sensitivity of 90% and specificity of 100%. Therefore, it simplifies patients' management preventing unnecessary biopsies [7]. Furthermore, the correct management of breast seroma cytological samples is critical for the diagnosis of implant-associated anaplastic large-cell lymphoma [8].

We report a unique case of HL, clinically presenting as inflammatory breast carcinoma diagnosed by FNA with rapid on-site evaluation (ROSE) and immunocytochemistry. This diagnostic procedure proved to be crucial for the patient's management for it prevented an unnecessary breast surgery and further delay of chemotherapy. It is also the first case of Hodgkin lymphoma with breast involvement and refractory disease, with an otherwise poor prognosis, successfully treated with monotherapy of brentuximab vedotin, the novel CD30-targeted immunotherapy.

## 2. Case Presentation

**Figure 1:**
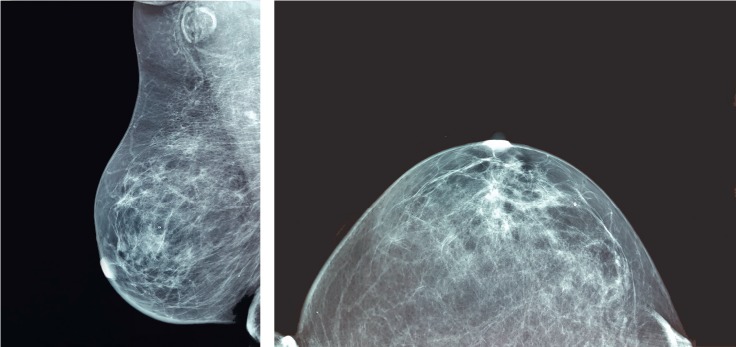
Mammography showing enlarged lymph node in the right axilla. No specific abnormalities were detected in the breast.

A 57-year-old woman presented with signs of inflammation, hardness, and lymphedema, with orange peel appearance of the skin of the right breast. A palpable mass of the right axilla preceded the breast lesion. It was gradually enlarging during a three-month period and did not respond to treatment with antibiotics. A mammography, performed when the axillary mass, was first observed, and it did not detect any abnormality in the breast, except of an enlarged lymph node in the axilla (Figure 1). Her medical history included hysterectomy for a mucinous borderline tumor of the right ovary four years ago.

**Figure 2:**
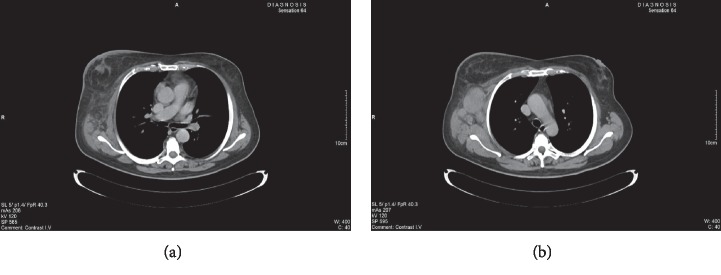
CT revealing breast lesion with partially ill defined borders, thickening of the breast skin, and lymphedema (a) and enlarged axillary lymph nodes (b).

Initially, a clinical diagnosis of inflammatory breast carcinoma was made and an FNA of the axillary lymph nodes was first attempted elsewhere. The cytology reported bloody material with few lymphoid cells and scant atypical epithelial cells, suspicious for malignancy. Immunocytochemistry was not performed. Following blind core biopsies of the breast did not reveal malignancy. The patient refused further investigation. A month later, she complained for further enlargement of the axillary mass and mild fever. At that time, clinical examination also showed palpable ipsilateral supraclavicular lymph nodes in the right region. A subsequent computed tomography (CT) scan confirmed the enlargement of axillary and supraclavicular lymph nodes. In addition, CT revealed a lesion with ill-defined border in the right breast considered suspicious for primary malignancy, skin thickening, and lymphedema (Figure 2). Her biochemical and full blood tests were within normal limits but showed an elevated red blood cell precipitation of 107 mm/h. The patient refused surgical biopsy of the axillary lymph node block; thus, she was referred to us for repeated FNA.

**Figure 3:**
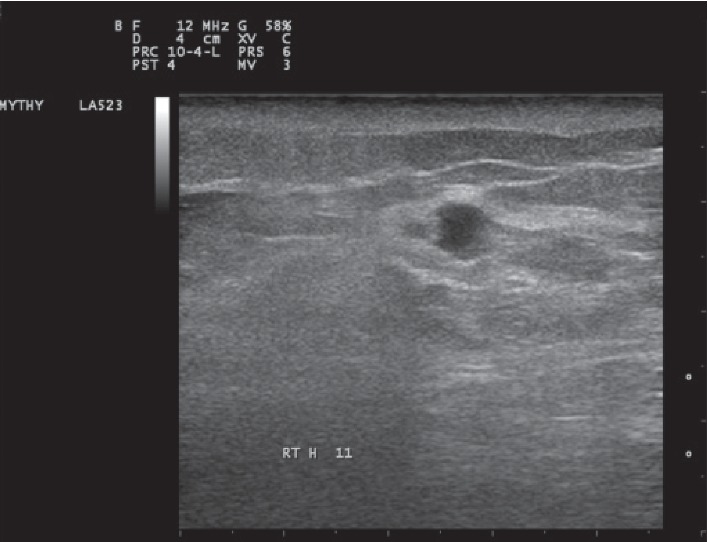
US revealing a small hypoechoic breast lesion with partially ill-defined borders and mild posterior acoustic enhancement.

By ultrasound (US), we observed in the right breast a hypoechoic, partially ill-defined lesion with mild posterior acoustic enhancement, measuring 8 mm in its maximal diameter. It was classified as BIRADS 4 (Figure 3).

**Figure 4:**
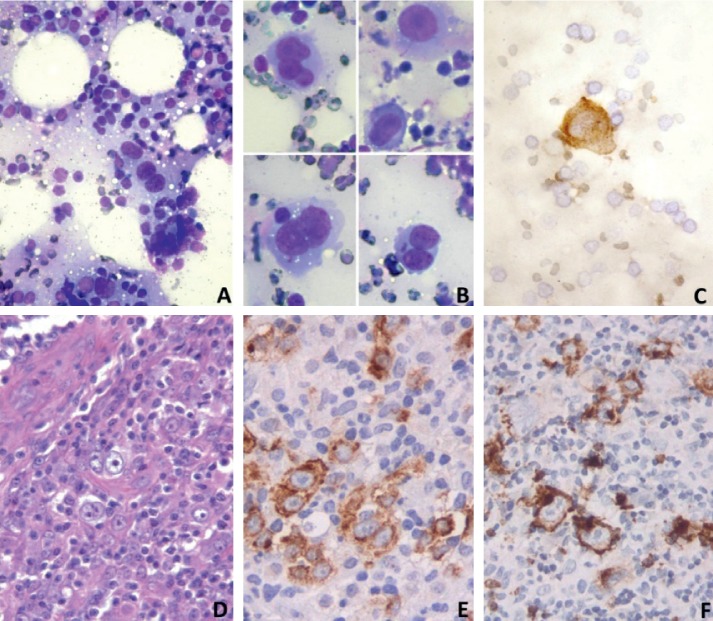
Cytology: (a) breast FNA showing scattered Reed–Sternberg and Hodgkin cells admixed with lymphocytes and eosinophils (Hemacolor stain, 20X); (b) Reed–Sternberg and Hodgkin cell variants (Hemacolor stain, 40X); (c) CD30 immunoreactive Hodgkin cell in cytology smear (ABC, 40X). Histology: (d) lymph node specimen showing Reed–Sternberg and Hodgkin cells in a classic milieu (H & E stain, 25X); (e) CD30 immunoreactive Reed–Sternberg and Hodgkin cells; typical Golgi pattern of chromogen distribution (25X); (f) Reed–Sternberg and Hodgkin cells with Golgi pattern immunopositive for CD15 (25X).

US-guided FNA targeted the breast lesion and the axillary lymph node block consecutively. FNA was performed with rapid on-site evaluation (ROSE) of the specimens using Hemacolor rapid staining (Merck, Darmstadt, Germany). The remaining slides were evaluated after Papanicolaou stain. The cytomorphology of the breast and axillary lesions was identical. Scattered large neoplastic lymphoid cells were observed among a reactive population of small lymphocytes and eosinophils. The neoplastic cells showed abundant faint basophilic cytoplasm and large nuclei with prominent nucleolus. A substantial number of them were binucleated with typical features of Reed–Sternberg cells and few were multinucleated. Immunostains were performed on some of the Hemacolor-stained slides. The neoplastic cells were immunopositive for CD30 and CD15 and negative for LCA. The latter was immunoreactive only in the small lymphocytes. Pan-keratin was negative. A nonepithelial neoplasm, consistent with HL, was reported. Following the unexpected cytological diagnosis, surgical biopsy of the axillary lymph node block was performed. A tissue specimen of aprox. 1.1 cm was forwarded for histology. Classical HL of nodular sclerosis subtype was reported, confirming the cytological diagnosis. The Hodgkin/Reed–Sternberg cells, in addition to CD30 and CD15 immunoreactivity, were positive for MUM1 and CD4 (approx. 50%). PAX5, CD20, CD3, and CD79a were negative. The small lymphocytes consisted of a mixed T (CD3 positive) and B (CD20 positive) population. Small CD4 positive lymphocytes focally formed rosettes around Hodgkin cells (Figure 4).

The disease stage was IV B with extranodal right breast involvement. The patient received ABVD regimen (doxorubicin, bleomycin, vinblastine, and dacarbazine). After two full cycles, she showed a satisfactory (more than 50%) reduction of the lymph nodes mass on CT. One month before completion of all ABVD cycles, the axillary mass was palpable again. She was restaged with CT, which showed a remarkable increase of the lymph nodes block, and a PET-CT showed increased fluorodeoxyglucose (FDG) uptake in the right axillary and breast region. The patient was considered primary refractory HL, and salvage chemotherapy with DHAP regimen (dexamethasone, cisplatin, and cytarabine) was given. After one cycle of DHAP with mild toxicity (grade II no febrile neutropenia), a new CT was performed to check for chemosensitivity in order to proceed to autologous transplantation consolidation. The CT scan showed no improvement, and a cycle of DICE (dexamethasone, ifosfamide, cisplatin, and etoposide) in combination with brentuximab vedotin (BV), a newly introduced anti-CD30 antibody-drug conjugate, was decided. Grade IV hematological toxicity was observed, and the patient was hospitalized due to febrile neutropenia, anemia, and pyelonephritis. After hospitalization for a week with fluid and antibiotics intake, granulocyte colony stimulating factor (GCSF) administration, and red blood cell and platelet transfusions, she gradually improved and was discharged from the hospital. The patient denied any further aggressive chemotherapy. The treatment continued with BV monotherapy at a dose of 1.8 mg/kg every three weeks. After four infusions, a CT scan showed total remission of lymphadenopathy, the breast lesion disappeared, and lymphedema diminished substantially. The above findings were also confirmed with a negative PET CT scan. The patient continued receiving nine more cycles of BV every three weeks. Therapy was completed after thirteen infusions in total. Repeated annual restaging with CT scan revealed no signs of relapse. After two and a half years of follow-up, the patient is free of disease, in good health, and the breast has turned to normal.

## 3. Discussion

Classic HL is a monoclonal neoplasm composed of Hodgkin/Reed–Sternberg cells admixed with a heterogeneous population of nonneoplastic immune cells usually arising in the lymph nodes [9]. Extranodal manifestation of Hodgkin lymphoma is less frequent than non-Hodgkin lymphoma. It can involve several organs mimicking other neoplasms or infectious processes [10]. Breast is an uncommon site affected by HL. According to a recent review, 19 cases of primary HL of the breast have been reported since the first description by Kueckens in 1928 [4, 11]. However, after a better search of the literature, we retrieved 5 additional cases [12–16]. Breast involvement in the process of disseminated or recurrent Hodgkin lymphoma has also been reported [5, 17].

Breast lymphoma presents as a palpable tumor with or without axillary lymph node involvement. Mammographic findings are not pathognomonic. In the majority of cases, breast lymphomas appear as round or irregular masses with well- or ill-defined margins, showing no calcifications or sclerosis. In a smaller proportion, they can appear as architectural distortion or as a combination of a mass with architectural distortion pattern. Rarely, mammography shows no abnormalities [2, 18]. In the present case, mammography did not show any specific findings in the breast except of the enlarged axillary lymph node.

US in breast lymphomas demonstrates mostly oval or round lesions with distinct or microlobular margins, hypoechoic or with a mixed echo pattern, and posterior acoustic enhancement. Some cases, significantly hypoechoic or anechoic lesions closely, mimick cysts [2]. However, in a recent multicentre study of a large number of hematopoetic malignancies involving the breast, most lesions show irregular shape, complex echo pattern, indistinct margins, and various posterior acoustic phenomena [18]. In general agreement, US has a higher sensitivity in detecting hematological malignancy of the breast compared to mammography [2, 18]. In our case, the US features are in agreement with the literature [2].

On CT, the findings are not specific overlapping with other entities as cysts, fibroadenomas, and malignant tumors [2]. In the present case, the identified breast lesion, although not specific, it was considered suspicious for primary malignancy.

Although imaging studies in cases of breast lymphoma can suggest malignancy, they are not helpful in differentiating lymphoma from carcinoma. Therefore, accurate diagnosis is based on biopsy [19].

Breast lymphoma can rarely present with extended lymphedema, stimulating inflammatory breast carcinoma. To the best of our knowledge, three cases of HL with breast inflammatory presentation have been reported so far [14, 15, 20]. Our case shows similarities with the previous reports regarding the clinical appearance. However, it is unique, because unlike these cases which were diagnosed by histology, the initial diagnosis was achieved by FNA further confirmed by histology.

The role of cytology in the diagnosis of breast NHL is well established [7, 21]. HL can also be diagnosed reliably by cytology and immunocytochemistry performed on cytospin or cell block material [6]. However, most of the breast Hodgkin lymphoma cases reported in the literature have been diagnosed by histology of the surgical specimen after lumpectomy or on core needle biopsy material.

This fact is related to the rarity of the entity and to the limited use of FNA as a diagnostic approach of breast lesions in routine practice lately. A recent review states that breast HL cannot be diagnosed by FNA. This can be attributed mainly to inadequate or inconclusive material, particularly in cases of nodular sclerosis with abundant collagenous stroma [4]. In another case, cytology was reported as suspicious for malignancy [17]. Hodgkin cells may simulate carcinoma, in paucicellular specimens lacking the full spectrum of the lymphoid cell population. That was the reason for misdiagnosis in the first FNA attempt in our patient, in which no immunostains for keratin or other markers were performed. Even in core biopsy, in the absence of typical Reed–Sternberg cells, the presence of accumulated epithelioid histiocytes and multinucleated giant cells may lead to erroneous report of granulomatous inflammation [20].

In only two cases of breast Hodgkin lymphoma, the initial diagnosis was suggested by FNA cytology [13, 22]. In one of them, immunohistochemistry was carried out on the cell block. In both, the final diagnosis was confirmed by histology [22]. In our case, ROSE procedure during the FNA proved to be a valuable diagnostic tool. Using ROSE, we were able to ascertain the specimen sufficiency and recognize the lymphomatous nature of the breast lesion, leading to direct FNA of the axillary lymph node mass. Therefore, we obtained adequate material for immunocytochemistry. In our case, the presence of typical Reed–Sternberg cells was the diagnostic clue. This is in concordance with the two previous reports [13, 22]. Immunopositivity of the Hodgkin cells for CD30 and CD15 in conjunction with LCA negativity further sustained the diagnosis. In addition, negative immunostain for pankeratin excluded the diagnosis of carcinoma. Keratin markers are useful in the differential diagnosis between HL and metastatic carcinoma in lymph node FNA. Furthermore, keratin positivity in a breast FNA showing large malignant cells among abundant lymphoid population can confirm the diagnosis of medullary carcinoma, which may pause diagnostic difficulties. Histological study, which followed, confirmed the unexpected cytological diagnosis, reporting a classical HL of nodular sclerosis subtype, commonly observed involving the breast [4, 5, 12, 14, 15, 17, 20, 22].

Our case represents a successful example of cytological diagnosis of Hodgkin lymphoma in breast, despite the misleading clinical presentation. This minimally invasive diagnostic approach proved crucial for avoiding an unnecessary major breast surgery, which would further delay chemotherapy. Instead, the patient underwent minor lymph node biopsy for histology.

The therapeutic management of our patient was also challenging because she developed relapsed/refractory disease, not responding to salvage chemotherapy regimens, and she experienced severe side effects. BV is a novel therapeutic agent for CD30-positive lymphomas, the first immunotherapy that was approved by the United States Food and Drug Administration (US FDA) for the treatment of Hodgkin lymphoma. The drug consists of a tubulin toxin (monomethyl auristatin E) conjugated on anti-CD30 monoclonal antibody, which binds to the CD30-expressing neoplastic cells. The conjugate inhibits microtubule formation prohibiting mitosis, leading to cell cycle arrest and apoptosis [23, 24]. BV is being investigated in the management of relapse/refractory Hodgkin lymphoma as monotherapy or in combination with chemotherapy regimens with promising results [25]. In addition, it has recently been incorporated in the treatment of newly diagnosed HL patients with encouraging results, at least in some patients [26]. Previous studies have also proved the importance of BV as maintenance in high-risk patients after autologous transplantation [27] and as a bridge to allogenic transplantation [28].

In our case, the administration of DICE in combination with BV was discontinued, because of severe toxicity resulting in patient's unwillingness to receive any further intensive chemotherapy. Eventually, monotherapy with BV proved to be effective, resulting in total remission and long-term disease-free survival. The treatment was well tolerated. According to the literature, monotherapy with BV results in complete response in 35% of patients with relapsed/refractory Hodgkin lymphoma, causing minimal side effects [25]. The role of BV has already been established in different settings in the management of patients with HL and is regarded a valuable tool in the treatment of the disease. Therefore, the role of immunohistochemical markers such as CD30, also applied in cytologic material, is of paramount importance for therapy.

In conclusion, Hodgkin lymphoma, although very rare, should be included in the differential diagnosis of breast lesions with inflammatory presentation. FNA assisted by ROSE and immunocytochemistry may serve as a primary minimally invasive procedure for a prompt diagnosis in order to avoid unnecessary surgical management and delay of chemotherapy. In addition, it is the first case in the literature of Hodgkin lymphoma, with breast involvement and refractory disease, with an otherwise poor prognosis, cured with monotherapy of the novel CD30-targeted antibody drug conjugate. This report highlights the key role of the pathologist, not only in the diagnosis but also in the decision for therapy in the new era of precision medicine.
